# Assessing Human Diet and Movement in the Tongan Maritime Chiefdom Using Isotopic Analyses

**DOI:** 10.1371/journal.pone.0123156

**Published:** 2015-03-30

**Authors:** Christina Stantis, Rebecca L. Kinaston, Michael P. Richards, Janet M. Davidson, Hallie R. Buckley

**Affiliations:** 1 Department of Anatomy, Division of Health Sciences, University of Otago, Dunedin, New Zealand; 2 Department of Anthropology, University of British Columbia, Vancouver, British Columbia, Canada; 3 Department of Human Evolution, Max Planck Institute for Evolutionary Anthropology, Leipzig, Germany; 4 Museum of New Zealand Te Papa Tongarewa, Wellington, New Zealand; ICREA at the Universitat Autònoma de Barcelona, SPAIN

## Abstract

The rise of stratified societies fundamentally influences the interactions between status, movement, and food. Using isotopic analyses, we assess differences in diet and mobility of individuals excavated from two burial mounds located at the `Atele burial site on Tongatapu, the main island of the Kingdom of Tonga (c. 500 - 150 BP). The first burial mound (To-At-1) was classified by some archaeologists as a commoner’s mound while the second burial mound (To-At-2) was possibly used for interment of the chiefly class. In this study, stable isotope analyses of diet (δ^13^C, δ^15^N, and δ^34^S; *n* = 41) are used to asses paleodiet and ^87^Sr/^86^Sr ratios (*n* = 30) are analyzed to investigate individual mobility to test whether sex and social status affected these aspects of life. Our results show significant differences in diet between burial mounds and sexes. Those interred in To-At-2 displayed lower δ^13^C values, indicating they ate relatively more terrestrial plants (likely starchy vegetable staples) compared with To-At-1 individuals. Females displayed significantly lower δ^15^N values compared with males within the entire assemblage. No differences in δ^34^S values were observed between sexes or burial mound but it is possible that sea spray or volcanism may have affected these values. One individual displayed the strontium isotopic composition representative of a nonlocal immigrant (outside 2SD of the mean). This suggests the hegemonic control over interisland travel, may have prevented long-term access to the island by non-Tongans exemplifying the political and spiritual importance of the island of Tongatapu in the maritime chiefdom.

## Introduction

The rise of stratified societies fundamentally alters access to certain foods by individuals based on their status [1,2], and the potentially complex interactions between diet, migration, and social status can greatly affect health [3]. In past populations, social status can be inferred by examining dietary differences with regard to access to certain foods, especially those considered ‘high status’ by a particular community [4]. Stable isotope analyses can be used to identify dietary differences between certain social groups, as identified by non-biological markers (such as grave goods and burial type) and sub-groups as determined by biological markers (age and sex) [5–7]. Isotopic analysis of tooth enamel grants the opportunity to explore movement in individuals from a skeletal assemblage and consider the effects of rank, status, and hegemonic control of territory on the mobility of individuals [8,9].

**Fig 1:**
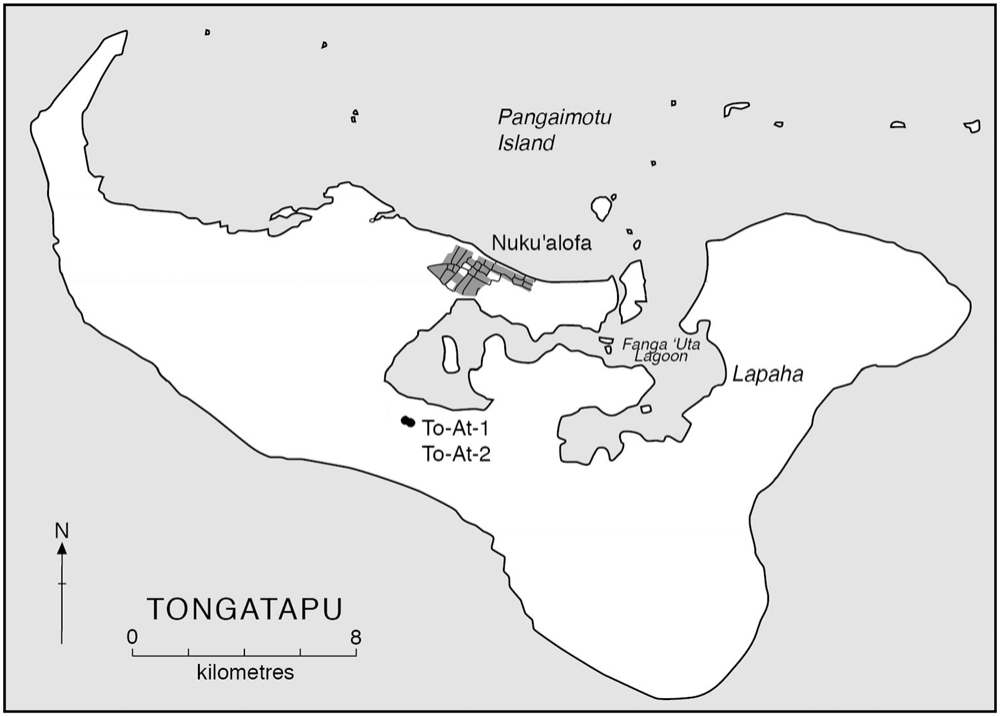
Map of Tongatapu. `Atele burial mounds (To-At-1 and To-At-2) labeled. Image courtesy of Geoffrey Clark.

In Polynesia (the area geographically confined within the rough triangle of Hawai`i, New Zealand, and Rapa Nui) many cultures are/were highly stratified [10–12]. The Chiefdom Period (c. 750–150 BP) was a time of hegemonic control of the Tongan maritime chiefdom [13]. During this period the Tongan paramount elite exerted their influence over neighboring islands, sending junior-ranking chiefs to outlying islands to maintain power and assure the flow of prestige goods (e.g., fine mats, feathers, sandalwood, barkcloth, canoes, and pottery) back to Tongatapu (*Sacred South*), the center of the Tongan chiefdom (Fig 1) [14–17]. During the Chiefdom Period, the basic societal frameworks of the complex Polynesian hierarchical culture were fully developed [17]. The concepts of rank and status, most likely long engrained in Polynesian society [18], gave the paramount chief (the *Tu`i Tonga*) the capability to exert the influence necessary for monumental architecture, sweeping landscape change, and steady extraction of surplus from the lower classes in the form of tributes in order to support these undertakings [12,13].

Like many cultures with a strict hierarchical political structure the importance of rank continued in death [19,20]. In the classic definition of Tongan burial mounds by archaeologist McKern [21], small, shallow mounds (*tanu’anga*) were used to inter commoners and larger mounds with a surrounding ditch (*fa’itoka*) were designated for chiefs and their retainers. *Langi*, the largest of mounds with large coral block inlays, were reserved for the Tu`i Tonga and his family.

The archaeological site of `Atele on the island of Tongatapu is invaluable for testing the validity of McKern’s mound classification [22]. Two burial mounds were excavated at `Atele in 1964. The first burial mound, (To-At-1), would be classified using McKern’s definitions as a *tanu’anga* because of its relatively shallow height of 80 cm. To-At-2 was taller (2.5 m high) and associated with a shallow encircling ditch, the classic form of a *fa’itoka*. Despite the difference in size, Davidson was critical of whether these mounds represented status-related burial places because of the lack of associated grave goods in either mound and the similarities in the method of interment and overall structure of the mounds [22].

Recent AMS dating places the interments of both mounds between *c*. 460–0 cal BP, which corresponds with the Chiefdom Period in Tonga (Table 1). These dates are markedly different from the original radiocarbon dating cited in Davidson’s report [22], which placed the burials within the Formative Period (1500–750 BP) of Tongan prehistory. The increased accuracy of modern dating methods [23] and expanded understanding of how marine reservoir offsets affect estimations [24] probably account for this discrepancy. Although the current radiocarbon range could place some of the burials within an early historic time frame (150–0 BP), this is unlikely for two reasons: both the Honorable Ve`ehala (Keeper of the Palace Records at the time) and current occupants of nearby villages had no recollection of these burials and, importantly, there were no artifacts indicative of European contact found in either mound [22].

**Table 1:** Radiocarbon dating results for `Atele burial mounds.

**Burial**	**Laboratory designation**	δ^13^ **C**	Conventional ^14^ **C Age (BP)**	**Calibrated age range cal BP (68.2% prob.)**	**Calibrated age range cal BP (95.4% prob.)**
**To-At-1/6**	Wk-38144	—-	278 ± 21	254–215	279 - -2
**To-At-1/34**	Wk-38145	—-	489 ± 22	323 - -2	460 - -2
**To-At-2/4**	Wk-38146	—-	232 ±23	256–7	281 - -1
**To-At-2/24**	Wk-38147	—-	220 ± 23	255–10	280 - -1
**To-At-2/26**	Wk-38148	—-	280 ± 22	248 - -2	284 - -2

AMS dating from Waikato Radiocarbon Dating Laboratory. The δ^13^C values were measured on prepared graphite using the AMS spectrometer due to the small size of the samples, and so the radiocarbon dates have been corrected for isotopic fractionation. However, the AMS-measured δ^13^C values can differ from the δ^13^C of the original material and are not listed. Dates are corrected for a lagoon reservoir using a ΔR value of 273 [24] and calibrated using the IntCal13 and Marine13 curves [142] in OxCal v4.2.3 [143].

The skeletal collection from `Atele (*n* = 129 individuals) is one of the largest and best preserved in Polynesia. This assemblage was first analyzed by Pietrusewsky in 1969 [25]. More recent bioarchaeological investigations of health [26] and activity [27] were conducted using the collection, neither of which found differences in disease prevalence or activity patterns between the mounds. However, Buckley [26,28] did find possible diet-related disease in the subadults of both `Atele mounds, increasing the importance of further characterizing diet in this population. Unfortunately, these studies are limited for interpreting differences in status, as status may not be reflected in disease prevalence or activity. Isotopic analyses, however, provides direct insight into diet and provenance. By studying the isotopic composition of human bone collagen, it is possible to directly test questions regarding the relative proportion of certain types of food in an individual’s diet (e.g., marine and terrestrial) [29,30].

A recent chemical analysis of stone tools from West Polynesia demonstrates direct evidence of the movement of prestige goods to Tongatapu from Fiji, Samoa, and as far away as the Society Islands (2500 km east of Tongatapu) [31]. Clark et al. [31] posit that the Tongan maritime chiefdom encouraged the establishment of specialized crafting sites that “formed important centers for the transmission of information, people, and material in prehistoric Oceania” (p. 10491), thus implying the movement of people as inferred through the movement of goods. However, examining the movement of people through strontium isotopic analysis of human tooth enamel [8,9] provides an opportunity to directly test inter-island contact and mobility in people contemporaneous with the Chiefdom Period lithics analyzed by Clark and colleagues.

Using the isotopic results from the bones and teeth of 45 individuals of known sex we aim to address three hypotheses. With no previous evidence of status-based differences, we test the hypothesis that the individuals from the two burial mounds, possibly representing two social classes, will have distinctly different diets as inferred from stable isotope analyses. We also compare diet and mobility between the sexes to consider possible sex-based sociocultural differences. We hypothesize there will be no differences between the sexes regarding diet, given the practice of communal meal-sharing between extended family members and members of the community in Tonga [32,33]. Given the long history of exchange between Tonga, Samoa, and Fiji [34], we also hypothesize that there will be evidence of high levels of immigration as reflected in the ^87^Sr/^86^Sr isotope values of the individuals interred in the mounds.

## Diet in Tonga

Post-contact European accounts highlight that Tongan diet, like most Polynesian diets, centers around a starchy staple plant food (“real food” or *kai*) such as taro (*Colocasia esculenta*), yam (*Dioscorea* spp.), and breadfruit (*Artocarpus altilis*), accompanied by side dishes (*kina*) such as animal flesh and grated coconut to provide flavor variety [32,35]. Most plants eaten in Tonga today and in the past were likely imported by the initial colonizers and include root crops and fruit and nut trees [12,36]. Terrestrial native plants are not part of the Tongan horticultural system but could be gathered in times of food scarcity, such as after extensive cyclone damage to gardens [12,37,38]. More commonly eaten are seaweeds and seagrasses, which are gathered today by women and children when foraging for shellfish and other inshore organisms in the lagoons and reefs [38]. The accompaniment to *kai* can consist of some sort of meat, most often some kind of seafood from the open ocean, reef, or lagoon [32]. Midden evidence from early sites (c. 2850–2650 BP) suggests shellfish and fish were the most common sources of protein in Tongan diet, though domesticated fauna were also represented in small quantities [39]. Pigs (*Sus scrofa)* and chickens (*Gallus gallus*) were present during the first European encounters to the “Friendly Islands.” During his first visit, Captain Cook found that the locals were familiar with dogs (*Canis familiaris*), despite the animals’ absence on Tongatapu [40]. Historical accounts report people of lower social standing consuming pigeons and rats [41].

Diet can be used as a correlate of social status in Polynesia. Esteemed luxury foods include fatty/greasy meats such as pig, dog, pelagic fish, and turtles [42,43]. However, the assumption that high status individuals ate proportionately more meat in their diet may not fully account for the sociocultural implications surrounding luxury foods in Polynesia. Regarding marine animals, pelagic fish and turtles were a rarity compared to reef fish and shellfish. Pigs occupied both the economic and ideological realms where the prestige associated with owning and gifting or tributing pigs was common, but there was an obligation for the upper classes to redistribute pork back to the populace during inclusionary feasting [44,45]. The upper classes of Polynesia were routinely tributed garden foods by the rest of society and, in Tonga, a large proportion of these tributes were kept by the upper class for consumption during feasts [41]. These foods could then be changed in complex/labor-intensive ways such as puddings or fermentation to increase the foods’ social value [32,43,44,46].

### Stable isotope analyses for dietary reconstruction in Polynesia

The analysis of carbon, nitrogen, and sulfur stable isotope ratios of bone collagen allows us to assess the diets of individuals within the last ten to twenty years of their lives [47]. Stable isotope analysis is increasingly used for understanding diet and several thorough reviews of the subject are available [48–50].

Carbon isotopic analysis (δ^13^C) can be used to differentiate between the consumption of marine and terrestrial plant foods, and give us a broad idea of what types of plants were eaten based on their carbon fixation pathways [51–54]. Terrestrial C_3_ plants display δ^13^C values approximately between -33 to -23 per mil (‰)[55]. While most marine autotrophs follow the C_3_ pathway, marine algae and cyanobacteria display relatively higher δ^13^C values compared with terrestrial plants because they utilize different sources of carbon such as oceanic bicarbonate [55,56]. Plants that use the C_4_ carbon fixation pathway typically display carbon values between -16 to -9‰ [55]. There are only two edible C_4_ plants of note in the tropical Pacific islands, sea grapes (*Cawlerpa racemosa*) and sugar cane (*Saccharum officinarum*), and neither are generally considered dietary staples in the wider prehistoric Pacific though a few populations appear to have eaten substantial amounts of these plants [57,58]. Thus, it is likely that higher carbon values in Pacific island humans can be associated with a larger marine component in their diet rather than increased ingestion of C_4_ plants. *Pandanus tectorius*, a commonly cultivated plant in Polynesia, follows another carbon fixating pathway (the CAM photosynthetic pathway) but will display carbon values indistinguishable from terrestrial C_3_ plants [55,59]. There are small amounts of trophic level spacing in animals; bone collagen from a carnivore is expected to have a carbon isotopic composition between 0‰ and 2‰ higher than the herbivores it consumes but this spacing is too small to use δ^13^C analysis to examine trophic level variation except in controlled studies [60].

Nitrogen stable isotope values (δ^15^N) provide information about the trophic level of an individual’s diet, with an animal displaying roughly 3 to 5‰ higher δ^15^N values compared with their prey [61–63]. Used in conjunction with carbon stable isotope ratios, δ^15^N values can also be used to understand the proportions of terrestrial and marine foods in a diet. Aquatic food webs (marine and freshwater) are more complex and typically contain more trophic levels and therefore higher δ^15^N values compared with terrestrial systems [52,53,57,62].

Sulfur stable isotope analysis for dietary reconstruction is not as well-established as carbon and nitrogen isotope analyses, but is emerging as a method for differentiating between terrestrial and marine food sources [64,65]. Marine seaweeds and plankton have extremely uniform δ^34^S values consistent with the δ^34^S range of sea-salt sulfates (approximately +20.99‰) [66]. Terrestrial and freshwater plants draw upon sulfur from a variety of sources and will show more variation than marine plants, typically within a range of -22 and +20‰ [64,67]. The sulfur composition of the underlying geological substrate and microbial processes in the soil are often the main contributors in terrestrial ecosystems [68], inspiring some researchers to used δ^34^S analysis as a tool for the geographic origin of human remains [69]. Trophic level shifts in δ^34^S also occur, although the enrichment is too small to detect except in controlled environments (1‰) [65].

Previous studies of Pacific island skeletal samples have examined the relationship between status and dietary isotopes. A study focusing on 99 individuals interred in the Polynesian outlier of Taumako (c. 300–750 BP) found sex- and status-related differences in δ^13^C and δ^15^N values. Specifically, males and higher status individuals from Taumako were eating foods from higher trophic levels, interpreted as valued meat products such as pelagic fish, marine turtle and pig [7]. A study of nine individuals, possibly elites, from Fiji (c. 200 BP) found no differences between the sexes [70]. The Fijians displayed δ^13^C and δ^15^N values consistent with a diet mostly consisting of terrestrial C_3_ plants with a significant contribution of fish, which was interpreted as consistent with a high-status diet, although no comparisons were made between these supposed elites and any commoner burials. From Teouma, the oldest-known cemetery in the tropical Pacific, significant sex-based differences in diet were found through isotopic analyses which were possibly related to sociocultural practices of food distribution or labor specialization at the site [71].

Nitrogen isotope analysis has been conducted previously on nineteen individuals from `Atele, and three of those nineteen were also analyzed for carbon and sulfur [72]. Unfortunately, the carbon, nitrogen, and sulfur analyses for the `Atele individuals by Leach et al. [72] were conducted in different laboratories, and so C:N, C:S, and N:S ratios cannot be used as reliable indicators of collagen integrity from the published data. In addition, most of the nitrogen results from `Atele individuals by Leach and colleagues analyzed whole bone powder rather than purified collagen (as this study used) and are thus doubly incomparable. As such, these previous analyses were not utilized, and all individuals with cortical bone present for sampling were analyzed in this study.

## Human Mobility in Tonga

The Polynesians were adept seafarers and navigators capable of regular long-distance travel in their outrigger canoes. In the Historic period (150 BP-) Tongans travelled to Samoa (880 km) and Fiji (750 km) regularly for trade, warfare, and spouse exchange [41,73]. Archaeological evidence [74] and the dispersal of Tongan loan words from Micronesia to Hawai’i [75] indicate these voyages were continuations of prehistoric contact. The prestige associated with materials exchanged suggests that the trade networks were controlled by and benefitting an elite group [12,41,76] but this does not necessarily mean that high-status individuals underwent the journeys personally.

Beginning sometime in the second millennium C.E., the Tongan maritime chiefdom involved the expansion of Tonga’s influence over Samoa, Fiji, and West Polynesian outliers. Junior-ranking members of the chiefly class would rule outlying islands, securing their position with intermarriage into local chiefly families and ensuring the movement of tribute back to Tongatapu [12]. The power of the maritime chiefdom was such that one 19^th^ century ethnography records that non-Tongans were not allowed to travel to Tongatapu, the center of the chiefdom, without escort [41].

Regarding sex-based differences in mobility, ethnohistoric accounts record the exchange of both husbands and wives between Tonga, Fiji, and Samoa [34]. This practice acted to reduce political unrest by removing possible competition with the highest leaders in each island group. For example, according to Tongan tradition the sister of the Tu`i Tonga was technically of higher rank than her brother. If she married a Tongan man her children would be of higher rank than the Tu`i Tonga’s and have grounds to vie for the paramount chief position after his death. However, if she married a Fijian her children (while still high-ranking) would have no claim to the position [34,77]. The marriages of high-status individuals are usually the focus of ethnohistoric records, but lower-ranking individuals could have practiced exogamy as well.

### Strontium isotope analysis to assess human mobility

A substantial and growing body of literature has used ^87^Sr/^86^Sr analysis to examine movement and migration in the Pacific islands [78–82]. As ^87^Sr/^86^Sr ratios of tooth enamel are comparable to the bioavailable strontium ratios of a given locale, ^87^Sr/^86^Sr analysis is used to determine where a person lived during childhood to understand individual movement [8,83]. Bone can be analyzed [84], but tooth enamel is more resistant to post-mortem diagenetic contamination and therefore considered more reliable [85].

The Kingdom of Tonga is an archipelago composed of two parallel, geologically distinct island chains. The western arc island chain, the Tongan Volcanic Arc, comprises high islands with little to no coral reef formation [86]. The lack of reefs greatly reduces the amount of marine life available around these islands. Basalt and andesite from the western island chain were used as base materials for a variety of tools and ash from the volcanic islands would periodically fall on the eastern islands, providing rich soil for horticultural pursuits [13]. The eastern island chain is nonvolcanic and largely composed of uplifted coral limestone [86]. The island of Tongatapu is part of the eastern island chain, and so is composed of coralline limestone, though the topsoil is largely volcanic tephra from the western islands [86]. The rich soil and extensive reef systems of the eastern islands supported the majority of the Tongan population throughout the archipelago’s occupation.

**Fig 2:**
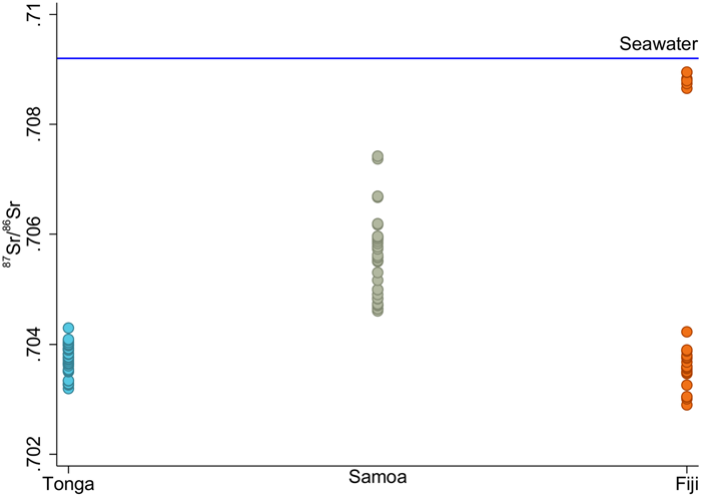
^87^Sr^/86^Sr isotopic compositions of Tonga, Samoan, and Fijian archipelagoes. Solid line is ^87^Sr^/86^Sr value of modern seawater (0.7092). Data from [87–90].

Geological baselines are available for comparison for this study [87–90] and show distinct isotopic compositions between archipelagoes (Fig 2). However, the use of geological data as proxies for understanding mobility may not yield accurate interpretations in coastal/island settings [91]. This is because the ^87^Sr/^86^Sr ratios of human tooth enamel are not wholly derived from geological isotopic compositions, but are reflective of the bioavailable strontium in an area, which is influenced by marine-based diets [92], atmospheric deposition, sea spray (saltwater spray from the waves or high winds), and marine-derived precipitation [93] in addition to the underlying bedrock. There are currently no published data for bioavailable ^87^Sr/^86^Sr ratios in West Polynesia or nearby Fiji. In the absence of bioavailable strontium baseline data, non-locals have been identified in this study by falling outside two standard deviations of the average ^87^Sr/^86^Sr of the combined assemblages of both mounds [9,78,94,95]. While somewhat arbitrary, this approach has proven effective in Pacific studies and other parts of the world to differentiate between locals and non-locals [79,80]. Sex-specific patterns of movement have not been found in other Pacific island mobility studies and no other studies have examined the possible relationship between mobility and social status in this region.

Originally used to examine paleoclimate in fossils [96], oxygen isotope analysis is another common method of examining movement [97–99]. Unfortunately, the averaging effect of oceanic rainfall creates homogeneous δ^18^O values across the tropical Pacific [100]. While some larger islands in Near Oceania have shown variation due to rivers (and thus some researchers have seen mixed success using δ^18^O in the Pacific [78,80,101]), detecting variation in Remote Oceania is unlikely. Many Remote Oceanic islands, Tongatapu included, have no running water and are solely supplied with freshwater by precipitation. Therefore, oxygen isotope analysis is not used in this study to examine human movement.

An unpublished PhD thesis has conducted strontium and lead isotope analyses on 16 individuals from the `Atele burial mounds [102]. While these individuals from To-At-1 and To-At-2 could have been invaluable comparative samples, Jaríc does not provide the individual burial numbers for the Tongatapu individuals using the original notation. Instead, Jaríc uses a personal identification system in her thesis without referring to the original system and so it is impossible to know which individuals were sampled. For this reason, all individuals from the `Atele burial mounds meeting the necessary criteria (below) were sampled for this study.

## Materials and Methods

All ages and sex estimations were determined by HRB. Seriation by degree of dental attrition was the primary method of assigning adults to age groups (Young, Middle, and Old adults) [103] because the differential preservation of postcranial material made age estimation using epiphyseal unions and pubic symphyseal degeneration difficult. Sex was estimated using the standards outlined in Buikstra and Ubelaker [104].

### Ethics Statement

Permission for these destructive analyses was obtained from the Tongan Royal Government. No permits were required for the described study, which complied with all relevant regulations. The `Atele skeletal collection is currently curated by the University of Otago Department of Anatomy (Dunedin, New Zealand).

### Carbon, Nitrogen, and Sulfur Analyses

Cortical bone fragments approximately 1200–1400 mg were isolated from each individual. Bones displaying pathological changes were excluded from sampling on the principle that changes in the metabolic pathways of the tissue as a result of disease may affect the isotopic values [105]. After sandblasting to remove surface impurities, collagen extraction and purification were carried out using a modified Longin method [106–108] at the Department of Anatomy, University of Otago (Dunedin, New Zealand). The samples were demineralized by soaking them in 0.5M HCL at 4°C followed by gelatinization in a 3.00 pH HCL solution in at 70°C for 48 hours. After filtering using a 5–8 μm Elkay Ezee mesh filter, the samples were ultrafiltered using Amicon Ultra-0.5 Centrifugal Filter Units with Ultracel-30 membranes [107] to remove peptides smaller than 30 kDa NMWL. The samples were frozen and then lyophilized for 48 hours.

Stable isotope analyses were conducted at the Department of Human Evolution, Max Planck Institute of Evolutionary Anthropology (Leipzig, Germany). Carbon and nitrogen isotope values were measured simultaneously using a Flash EA 2112 coupled to a DeltaXP continuous-flow isotope-ratio-monitoring mass spectrometer. Sulfur stable isotope composition was measured by combusting the samples in SO and SO_2_ gas in a HekaTech EuroVector elemental analyzer coupled to a Delta V Plus mass spectrometer. Repeated measurements of working standards EVA-0009 (methionine), SRM 1577b (bovine liver), IAEA-N-1 and-N-2 (ammonium sulfate), IAEA-CH-6 (sucrose), and IAEA-CH-7 (polyethylene) were interspersed throughout the archaeological samples to correct the carbon and nitrogen isotope data. For sulfur, IAEA-S-1 (silver sulfide), IAEA NBS-127 (barium sulfide), IAEA-SO-5 (barium sulfide), SRM 1577b and IVA-001 (casein protein) were interspersed.

Collagen integrity for carbon and nitrogen analyses was determined using %C by weight, %N by weight, and C/N ratios [109–111]. Collagen samples displaying C/N ratios between 2.9–3.6, %C values between 15–47%, and %N values between 5–17% were considered well preserved. Samples displaying a C:S ratio of 600 ±300, N:S ratio of 200 ±100, and a %S by weight of 0.15–0.35% were considered well-preserved [64,112]. Deviation from any of these criteria was cause for exclusion from statistical analyses and interpretations. All dietary stable isotope ratios are reported in delta (δ) notation ([R_sample_/R_standard_]-1)x 1000, where R is the ^13^C/^12^C or ^15^N/^14^N and standardized to the international references standards for carbon and nitrogen (Vienna Pee Dee Belemnite [VPDB] and Atmospheric Nitrogen [AIR], respectively).

### Strontium Analysis

Enamel samples were prepared and analyzed for strontium isotope ratios at the Department of Human Evolution, Max Planck Institute of Evolutionary Anthropology (Leipzig, Germany). The crown of each tooth (premolars or second molar) was sandblasted to remove the outer layer. A small piece of enamel was cut from the tooth using a dental rotary tool. Any dentine attached to the sample was ablated with a diamond-tipped engraving cutter. After being sonicated with 1 mL acetone and rinsed three times with water, samples were purified using the ion exchange method presented by Deniel and Pin [113] at the Department of Human Evolution, Max Planck Institute of Evolutionary Anthropology (Leipzig, Germany). The samples, SRM 1486 (bone meal), and blanks were analyzed using a Thermo Fisher Neptune plasma ionization multicollector mass spectrometer (PIMMS). Repeated measurement of international standard SRM 987 (bone meal) was used to ensure accuracy of data, and samples were adjusted using the published value of SRM 987, 0.710240 [114,115]. Isotope dilution analysis was used to obtain the strontium concentration (reported in ppm). A concentration calibration line was created by running the internal standards in each batch at three concentrations, 100 ppb, 400 ppb, and 700 ppb. Signal interference was corrected by measuring ^87^Rb, ^83^Kr, and ^82^Kr. Instrumental mass bias was normalized by measuring for ^88^Sr and using the natural ^88/86^Sr ratio of 8.375209.

## Results

Demographic, sampling, and isotopic data are presented in S1 Table. All 41 of the collagen samples reached the collagen quality indicators described above for carbon and nitrogen stable isotope analysis and were therefore included in the dietary interpretations. Although there was one outlier (2 SD) in terms of carbon isotope composition, To-At-2/33, no individuals were excluded from the statistical analyses. For sulfur stable isotope analysis, five collagen samples had insufficient collagen, but the remaining 36 bone collagen samples analyze for δ^34^S reached the collagen quality indicators described above for sulfur. Thirty-two individuals had teeth available for strontium isotopic analysis. Two of the 32 samples did not display any detectable strontium and were excluded. There was only one outlier outside two standard deviations of the mean population ^87^Sr/^86^Sr, To-At-1/09 (a female). A summary of δ^13^C, δ^15^N, δ^34^S, and ^87^Sr/^86^Sr isotopic results is presented Table 2.

**Table 2:** Summary statistics of isotopic results for the `Atele humans by sex and burial mound.

	δ^13^ **C**			δ^15^ **N**			δ^34^ **S**			^87^ **Sr/** ^86^ **Sr**		
	**mean**	**SD**	**n**	**mean**	**SD**	**n**	**mean**	**SD**	**n**	**mean**	**SD**	**n**
**Overall**	-17.6	0.8	41	9.3	0.6	41	14.5	1.7	36	0.7088	0.0001	30
**To-At-1**	-17.3	0.7	16	9.3	0.6	16	14.7	1.5	13	0.7088	0.0002	13
**To-At-2**	-17.8	0.8	25	9.2	0.6	25	14.5	1.8	23	0.7086	0.0001	17
**Females**	-17.8	0.5	23	9.0	0.6	23	14.7	1.5	21	0.7088	0.0001	21
***To-At-1***	-17.5	0.6	9	9.1	0.6	9	14.9	1.6	8	0.7088	0.0002	8
***To-At-2***	-18.1	0.2	14	9.0	0.4	14	14.6	1.5	13	0.7088	0.0001	13
**Males**	-17.3	1.1	18	9.7	0.6	18	14.3	1.9	15	0.7088	0.0001	9
***To-At-1***	-17.1	0.7	7	9.5	0.6	7	14.4	1.5	5	0.7088	0.0001	5
***To-At-2***	-17.5	1.1	11	9.5	0.7	11	14.3	2.1	10	0.7088	0.0001	4

To confirm that any differences between the burial mounds are not a result of varying proportions of males and females or age cohorts, Pearson’s chi-squared tests were computed. The proportion of males and females did not differ by burial mound, χ^2^(1, *n* = 41) = 0.001, *p* = 0.987. There were differences in the proportions of age categories (Young, Middle, and Old) between burial mounds, χ^2^(2,*n* = 32) = 6.16, *p* = 0.046. Examination of the demographic information (S1 Table) reveals that although there are similar numbers of young adults (8 individuals in To-At-1 and 7 in To-At-2) and middle-aged adults (5 in To-At-1 and 6 in To-At-2), old adults are only present in To-At-2.

**Fig 3:**
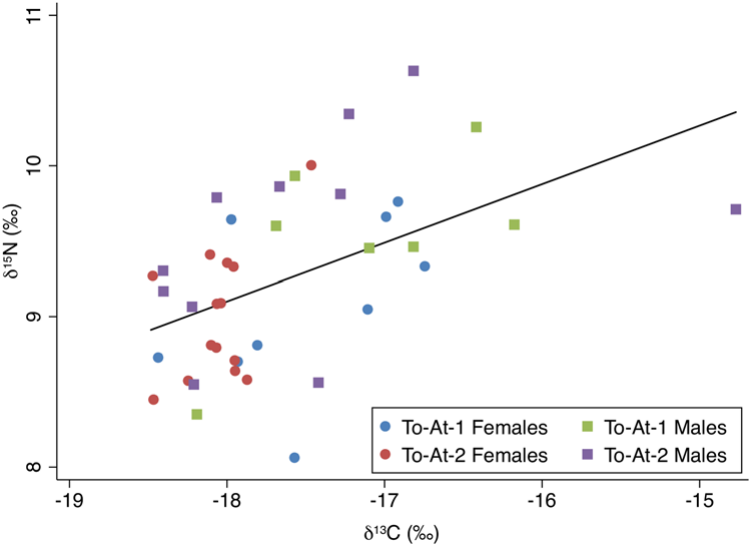
δ^13^C and δ^15^N values plotted by sex and burial mound with fitted regression line (y = 0.40x + 16.3, *p*<0.001).

**Fig 4:**
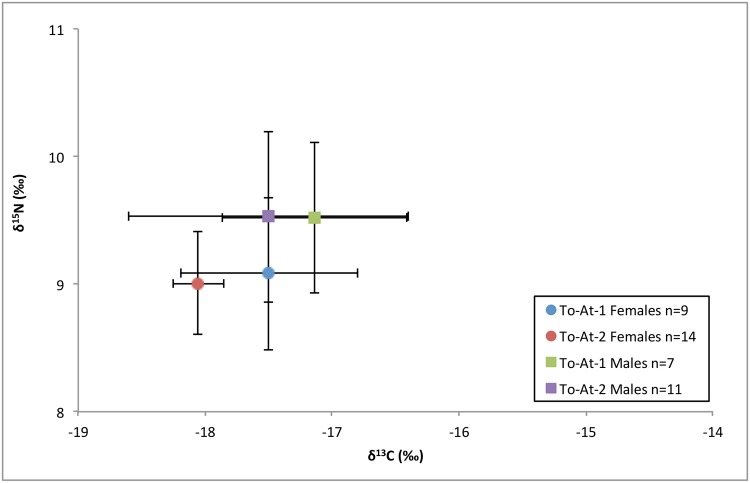
Mean (± 1SD) δ^13^C and δ^15^N results for sex and burial mound.

**Fig 5:**
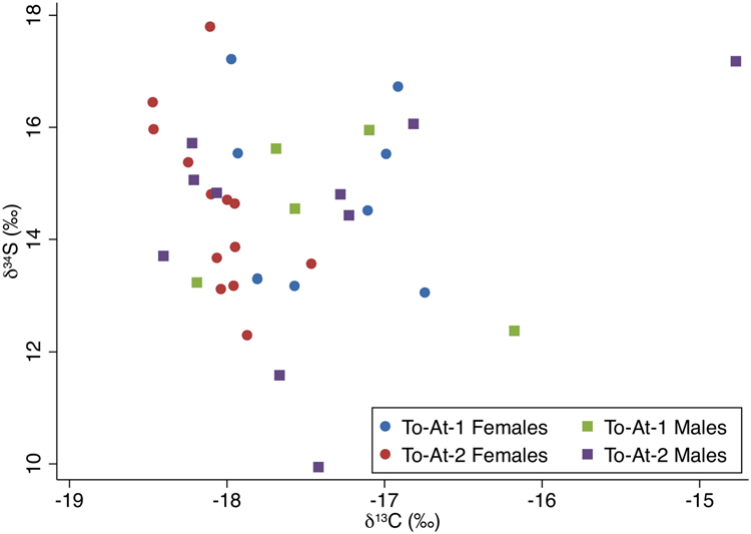
δ^13^C and δ^34^S values plotted by sex and burial mound. The δ^34^S outlier (To-At-2/27A) is labeled.

**Fig 6:**
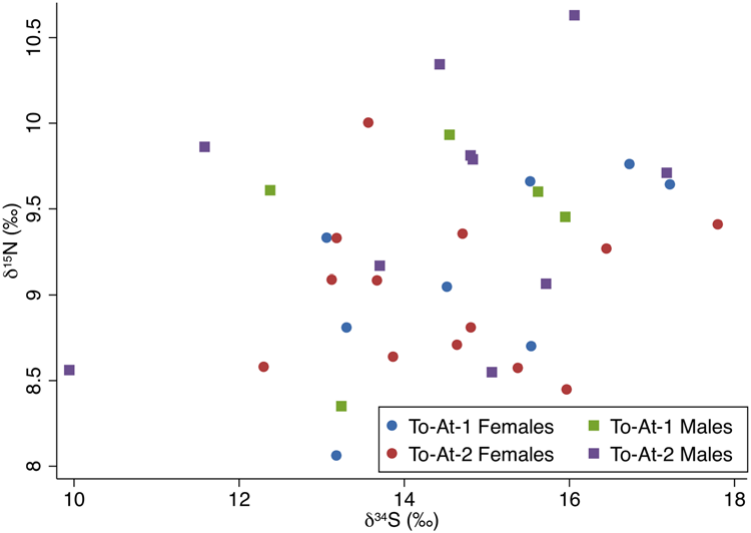
δ^34^S and δ^15^N values plotted by sex and burial mound. The δ^34^S outlier (To-At-2/27A) is labeled.

**Fig 7:**
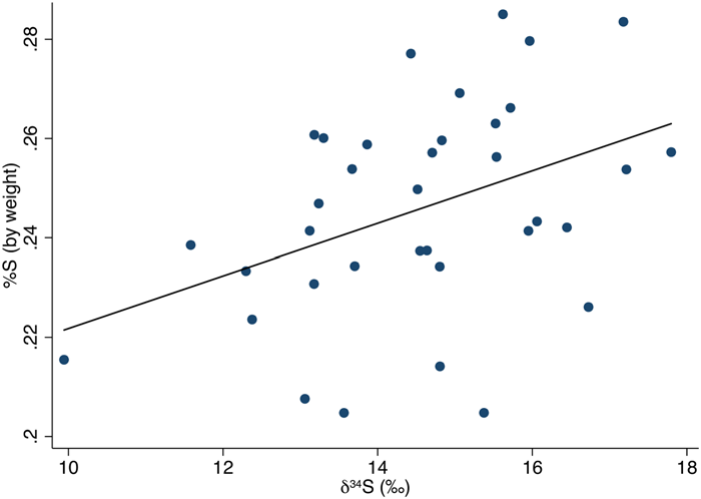
δ^34^S values and %S by weight with fitted regression line (y = 0.005x + 0.169, *p* = 0.028).

The carbon and nitrogen isotopic results are displayed on Figs 3 and 4. The carbon and nitrogen isotopic values of all individuals were strongly correlated, *r*(39) = 0.50, *p*<0.001. The δ^34^S results in relation to the carbon and nitrogen isotope results are plotted on Figs 5 and 6. Sulfur and carbon were not correlated, *r*(34) = -0.75, *p* = 0.543, nor were sulfur and nitrogen, *r*(34) = 0.03, *p* = 0.824. There was a significant correlation between δ^34^S and %S wt, *r*(34) = 0.27, *p* = 0.028 (Fig 7).

For δ^13^C, Levene’s test indicated unequal variances (*F* = 3.73, *p* = 0.02). A Kruskal-Wallis test indicated no differences between males and females regarding δ^13^C (*Z* = -1.58, *p =* 0.115). There were significant differences between burial mound regarding δ^13^C (*Z* = 2.70, *p =* 0.007), with To-At-1 individuals displaying higher values. There were no differences between the three adult age cohorts (young, middle, and old) regarding δ^13^C (*Z* = 0.93, *p* = 0.154).

The δ^15^N values were subjected to a factorial ANOVA with sex and burial mound as the independent variables. There were significant differences in δ^15^N values between sexes, *F*(1,40) = 2.22, *p* = 0.011, with males displaying a higher mean. The δ^15^N means between burial mounds were not significant, *F*(1,40) = 0.01, *p* = 0.841. The interaction effect was non-significant, *F*(1,40) = 0.02, *p* = 0.827. An ANOVA showed no significant differences between the age cohorts regarding δ^15^N, *F*(2,31) = 2.66, *p* = 0.0867.

Regarding δ^34^S values, a factorial ANOVA showed no significant results between sex, *F*(1,35) = 1.20, *p* = 0.534, or burial mounds, *F*(1,35) = 0.21, *p* = 0.793. The interaction effect was non-significant, *F*(1,35) = 0.17, *p* = 0.813. There were no significant differences between the age cohorts, *F*(2,26) = 5.10, *p* = 0.180.

**Fig 8:**
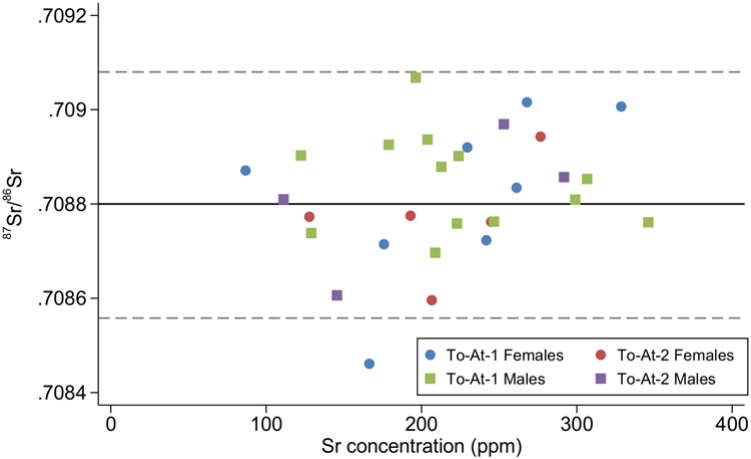
^87^Sr/^86^Sr values plotted by sex and burial mound. Population mean (solid line) and ± 2SD (dashed line) represented.

**Fig 9:**
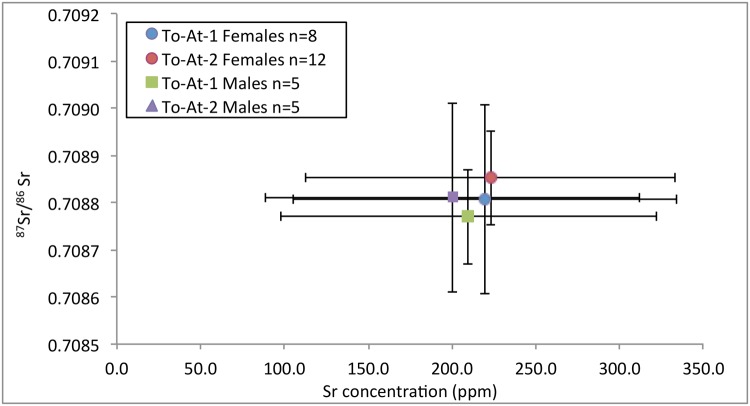
Mean (± 1SD) ^87^Sr/^86^Sr ratios for sex and burial mound.

The ^87^Sr/^86^Sr values are displayed on Figs 8 and 9. There were no differences regarding ^87^Sr/^86^Sr between sex *F*(1,29) = 0.56, *p* = 0.459, or burial mound *F*(1,29) = 0.37, *p* = 0.546. The interaction effect was non-significant, *F*(1,29) = 0.01, *p* = 0.905. There were also no differences regarding ^87^Sr/^86^Sr between age categories, *F*(2,23) = 0.67, *p* = 0.522.

## Discussion

**Fig 10:**
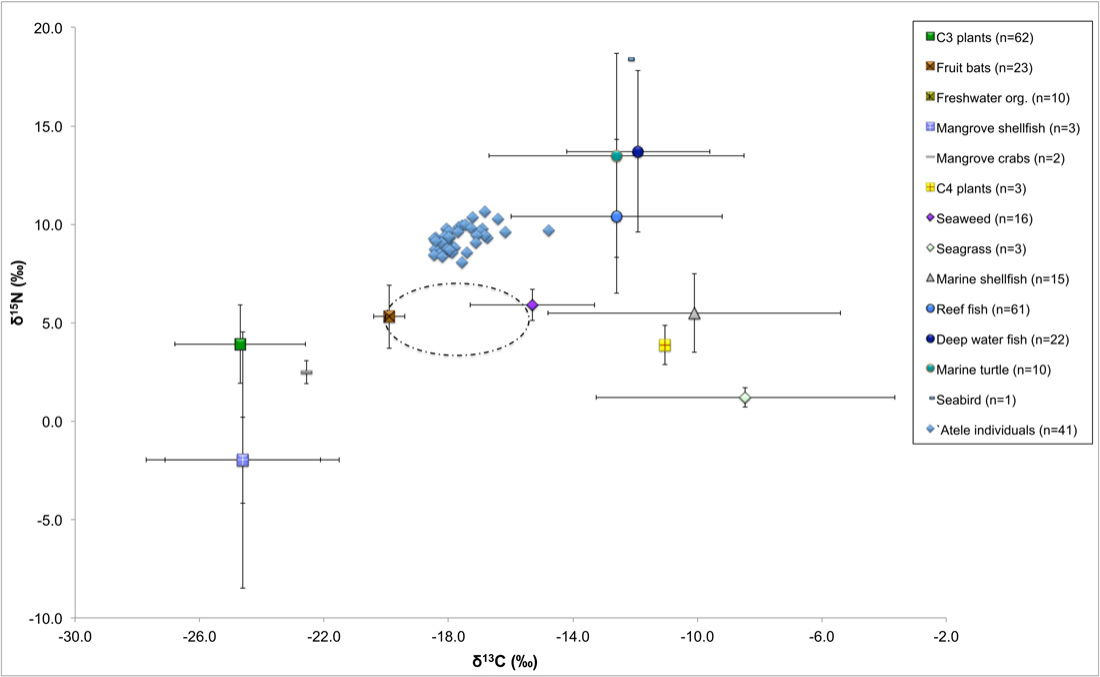
The `Atele δ^13^C and δ^15^N values compared to dietary baseline data from previous studies [71,82] Modern δ^13^C values have been corrected for the Suess effect, but are otherwise unaltered. The dashed circle indicates the diet of the individuals once corrected for trophic level, 1‰ for δ^13^C and 4‰ for δ^15^N values.

The correlation between carbon and nitrogen values may suggest that the differences in diet between individuals is a result of the different proportions of marine and terrestrial foods eaten [116]. There would be a lack of positive correlation if the population relied mainly on terrestrial and marine foods of the same trophic level or a single protein source [7,116]. Instead, the dietary trend suggests the entire population generally relied on marine animals and terrestrial plants. This trend can be observed when plotting the carbon and nitrogen isotopic values of the `Atele individuals to dietary baselines acquired from modern and archaeologically-derived tropical Pacific plants and animals [71,82](Fig 10). The bone carbon and nitrogen values of the fruit bat plotted on Fig 10 represent an animal consuming an entirely terrestrial diet. The human values, when adjusted for trophic level, fall between terrestrial plant foods and fish (reef and deep water).

A positive correlation between δ^13^C and δ^15^N values has been observed in other late prehistoric Pacific sites [7,57] and fits with ethnohistoric accounts of Polynesian diet, where starchy root vegetables such as taro (*Colocasia esculenta*) and yams (*Dioscorea* spp.), fruit trees such as bananas (*Musa* spp.), and nuts such as Tahitian chestnuts (*Inocarpus fagifer*) formed the base of subsistence. The stable isotope results of the `Atele individuals suggest these foods were accompanied by marine foods. Marine foods are largely collected from the nearby reef and lagoon environment in modern Pacific cultures, though open ocean species are also another important source of marine protein [32].

### Dietary differences in the mounds

The most significant findings to emerge from this study are the dietary differences between the mounds. With significantly higher δ^13^C values in burial mound To-At-1 compared to To-At-2, we fail to reject our first hypothesis. These findings are contrary to previous bioarchaeological studies which found no evidence for differences between the mounds as evidenced by disease [26,28] or activity [27]. There are several possible interpretations.

It is possible that the dietary differences between mounds are due to the differences in proportions of adult age groups. Old-aged adults are only present in To-At-2, and if older individuals were consuming less terrestrial foods compared to younger adults then that could be the cause of the burial mound differences. However, there were no significant differences between the age groups regarding paleodietary isotopic values and so it would seem age differences cannot explain the mound dietary differences.

Another possibility is the temporal difference between mound use. To-At-1 appears to have been used for interment between 278–479 ±22 BP, while To-At-2 was used between 220–280 ±23 BP. Though there is some overlap, To-At-1 was used at an earlier segment of the Chiefdom Period. Food procurement strategies can never be assumed to be static and it is possible that those interred in To-At-2 relied more on terrestrial plant foods not because they were accorded these foods due to higher status, but had access to better-established gardens with higher, more consistent yields. While temporal differences remain a possibility, the political environment of the period encompasses both mounds’ ranges of use.

It is also possible that the dietary differences between mounds are related to the different level of prestige accorded to certain foods in the tropical Pacific. While no Polynesian meal is truly a meal without starchy root vegetables, the consumption of large proportions of horticultural plants (especially when fermented or prepared into pudding) is associated with high prestige [32]. As such, starchy root vegetables played a key role in tribute to the Tu`i Tonga and other nobility during ceremonies [41]. Despite the low visibility of these low-protein foods in isotopic analyses obfuscating the true contribution of these plants to diet [51], the individuals of To-At-2 seem to have consumed more starchy root vegetables than the individuals of To-At-1. Initially, these results may be counter-intuitive given the status associated with certain types of animal foods. Greasy/fatty foods are arguably accorded an even higher status than labor-intensive puddings and fermentations, especially pork [44]. While pigs may have had increased status associated with their ownership or consumption, the relative abundance of lower-status animal protein, relative scarcity of high-status animal protein, and a culture of pork redistribution may suggest that Tongan nobility would not necessarily eat significantly more animal protein despite the status associated with certain animals.

The arguably small differences between mounds are likely reduced because many foods in the Pacific, regardless of associated status, are isotopically similar [7,71]. For example, a meal of roasted taro and raw coconut would display the same isotopic composition as the higher-status taro pudding with grated coconut cream as an emollient. Another confounding factor may be the cultural practice of burying servants with the chiefs in chiefly burial mounds although these servants were not necessarily eating the same foods as their chiefs [21].

It must be addressed that social status in Tonga is a continuum rather than discrete units. It has been said that no one in Tonga is the same rank as another [77], and the complex system regarding authority and ceremonial rank amongst individuals based on factors such as lineage, age, gender, and birth order complicates understanding status in the context of the discrete archaeological units used in this study: the two burial mounds. The burial mound classification created by McKern [21] defined three types of mounds, but this classification system does not take into consideration the complex heterarchy present in Chiefdom Period Tonga. It is entirely possible that both mounds are “commoner” mounds as Davidson concluded with no archaeological evidence suggesting status differences [22]. Instead, the individuals interred in To-At-2 may be generally of higher status such as skilled workers or retainers to a chief. This may also explain why the differences are small (though still significant).

### Sex-based dietary differences

Females displayed significantly lower δ^15^N values compared with males within the entire assemblage. The isotopic evidence supports the hypothesis that males and females were consuming different diets. This is not necessarily surprising as even the most egalitarian societies tend to show age- and sex-based differences regarding wealth, power, and access to foods [3,117]. However, it is important to understand the extent and type of differences in regards to diet as sex-based dietary differences have not been thoroughly addressed in Polynesian ethnohistoric literature, outside of island-specific taboos.

The δ^15^N differences between the sexes imply that females tended to eat foods from a lower trophic level compared with males, regardless of burial mound. Similar differences have been observed in Taumako, a Polynesian outlier and were interpreted as an indication of males having greater access to high status foods [7]. In Tonga, these differences could be attributed to sociocultural patterns of food allocation and consumption related to sex. Sisters are granted higher social status than their brothers in Tonga [77,118]. While this rarely translated to increased power or agency outside of ritual honors regarding births, weddings, and funerals [119], deference may have been given to women during mealtimes. Animal protein has been noted as restricted or controlled for women and may have repercussions on overall health [120]. In many parts of Polynesia, women were forbidden from eating certain animal foods [44] although evidence for this practice could not be found in Tongan ethnographic or historical literature.

Although both females and To-At-2 individuals display lower δ^13^C values than their comparative groups (though the difference between the sexes was not significant), the dietary discrepancies between the sexes may have less to do with social status (as the differences between the burial mounds might indicate) and more with how readily available foods are to certain individuals where varying forms of cultural pressures may result in near-identical archaeological patterns in the classic problem of equifinality [121,122]. Modern ethnographic studies have shown that Polynesian men are generally the fishers [32,123], and snacking between meals is a possible scenario that would cause males to display isotopic compositions reflective of higher trophic level and increased marine proportion [124]. Many fish and shellfish can be eaten raw on the beach or in the ocean [125]. However, it is often the task of women and children to gather lagoon and reef organisms in the Pacific, and so it is just as likely that they were snacking [125].

In modern Oceania, while maritime exploitation past shores and reefs is almost always within the male sphere, land-based food production can fall as a male-dominated or a female-dominated domain of labor [125–128]. In early historic Tonga, women were reportedly freed from “heavy” work and the female domain of labor was restricted to household duties (raising children, cleaning) and the production of wealth objects such as mats and barkcloth [129]. Ethnohistoric accounts support the concept that “boys go and girls stay” near or in the home [130]. Mariner, an English ship’s clerk who was stranded in Tonga between 1806 and 1810 wrote about the division of labor:

“It seems to be a peculiar trait in the character of the Tonga people… that they do not consign the heaviest cares and burdens of life to the charge of the weaker sex; but, from the most generous motives, take upon themselves all those laborious or disagreeable tasks which they think inconsistent with the weakness and delicacy of the softer sex. Thus the women of Tonga, knowing how little their own sex in other islands are respected…seldom associate with foreigners.” [41], p. 211

William Anderson, the surgeon’s mate on Captain Cook’s second voyage, also commented on the relative ease of Tongan women:

“The employment of the women is of the easy kind and for the most part such as may be done in the house. The province allotted to the men is as might be expected far more laborious and extensive than that of the women. Agriculture, Architecture, Boat building, Fishing and other things that relate to Navigation are the objects of their care.” [131], pp. 932–3

Furthermore, it has been argued that a united Tongan chiefdom gave men some degree of freedom from preparing for intertribal conflicts [129], thus increasing the amount of time they could spend in food procurement and “freeing” women from that burden. However, internecine conflict over control and distribution of goods, land, and prestige still influenced Tongan life during the Chiefdom Period [13,132]. We find the theory of prehistoric Polynesian women not contributing to horticultural production or tending of the earth ovens suspect. The women observed by Mariner and Anderson may have been of high status and thus not normally involved in manual labor anyway [133].

### δ^34^S values

Contrary to the carbon and nitrogen results, the δ^34^S values showed no significant differences between the burial mounds or sexes. There is one individual outside two standard deviations, To-At-2/27a, a middle-aged male. His carbon and nitrogen isotopic values are within the population averages. As δ^13^C and δ^15^N are correlated but the δ^34^S values are not correlated to the δ^13^C or δ^15^N values, the sulfur results might not be related to marine/terrestrial consumption. All samples with sufficient collagen for sulfur analysis displayed acceptable ranges of %S, C:S, and N:S. However, sulfur analysis is still a relatively unexplored avenue for paleodietary research compared to carbon and nitrogen analyses. From the statistically significant positive correlation between the δ^34^S values and %S, we believe the effect of sea spray or underlying geological formations rich in ^34^S may have affected the δ^34^S isotopic compositions of the bones [65,72,134].

The relationship between δ^34^S and %S has only been explored in one other isotopic study in Taumako, Solomon Islands [7]. The statistically significant negative correlation between δ^34^S and %S found in the Taumako population was interpreted as possibly resulting from diagenetic alteration from the effects of volcanism. Like Taumako, the topsoil of Tongatapu is largely volcanic ash (although Tongatapu itself is not volcanic: the volcanic islands to the west contribute topsoil) [86]. However, in the Taumako study, the correlation between δ^34^S and %S was negative; in this study, the correlation is positive. Given the small size of Tongatapu and large lagoon nearly bisecting the island sea spray is a possible contributor, although sea spray also does not satisfactorily explain the positive correlation. Although some success has been found examining populations from larger islands [135], the combined effects of sea spray and the local soil environment seem to leave δ^34^S analysis an inadequate tool for paleodietary reconstruction in many areas of the Pacific [71,82].

### Mobility in Tongatapu

**Fig 11:**
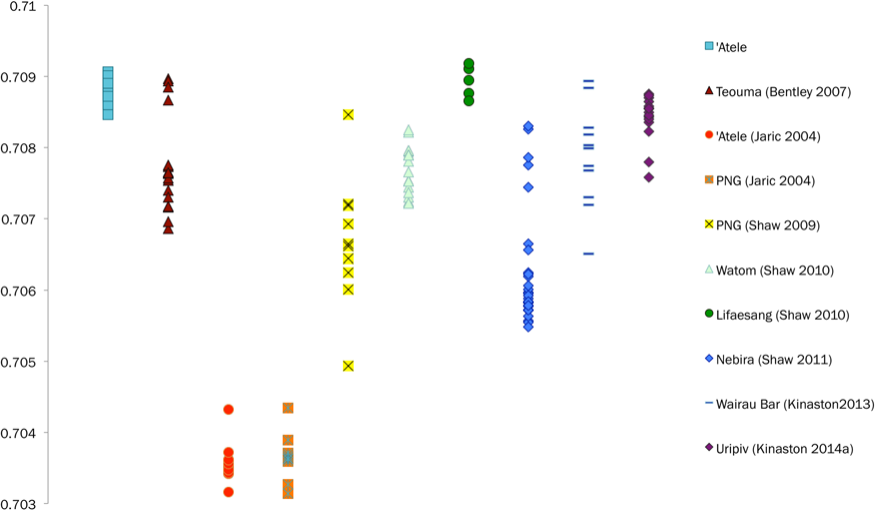
The `Atele ^87^Sr/^86^Sr ratios produced in this study compared to the ^87^Sr/^86^Sr ratios from previous prehistoric Pacific studies [78–82,101,102] Note the lower ^87^Sr/^86^Sr ratios by Jaríc [102].

When comparing our ^87^Sr/^86^Sr results to the strontium data from Jaríc’s PhD thesis [102], the tooth enamel ratios are completely different with no overlap; a cursory examination of ^87^Sr/^86^Sr ratios collected throughout the prehistoric Pacific reveals that Jaríc’s data are much lower than any other ratios produced in prehistoric Pacific individuals (Fig 11). Without burial numbers in Jaríc's thesis is it impossible to know for certain that we analyzed the same burials, but since every `Atele individual meeting our sampling criteria was analyzed in this study, it is unlikely that we did not sample many or most of the same individuals. Though a review of Jaríc’s methodology section does not reveal any outstanding errors, it can be safely assumed that, with ^87^Sr/^86^Sr ratios far different from any other values found in the Pacific, there may have been some issues surrounding Jaríc's methodology.

With only one outlier two standard deviations from the mean, most of the `Atele population probably spent their childhood locally. There is no difference in mobility as interpreted through ^87^Sr/^86^Sr between the two burial mounds or between the sexes. Although trade and other short-term interactions doubtlessly occurred within the nearby archipelagos, non-Tongans either did not live permanently on Tonga or were given different burial treatment and not interred in these two burial mounds.

Based on these analyses, we can infer the location of two points in these peoples’ lives: where they spent their childhood (using ^87^Sr/^86^Sr analysis) and their place of death. Inter-island movement during their lifetime cannot be determined. It is possible that, with the exception of To-At-1/09, everyone examined was born and died on Tongatapu. It is also possible they spent their lives after childhood travelling between islands before finally coming to rest back in their homeland. Trade and exchange between Tonga and the nearby island groups of Samoa and Fiji is recorded in historic times [41] and assumed to have occurred throughout occupation [74]. Clark and colleagues’ research demonstrates that Tonga was a center of prestige goods movement during the Chiefdom Period [31], so why do we see so little individual movement as demonstrated by ^87^Sr/^86^Sr analysis of tooth enamel? From the current analyses, it would appear that while non-local prestige goods were transported to the center of the Tongan chiefdom, either the people returning to Tongatapu with these goods were born on Tongatapu, or they were nonlocals from other archipelagoes who were not staying on Tongatapu. This second possibility must be made with the caveat that non-locals may have been buried in other ways and not accorded a ‘Tongan-style’ burial.

Both scenarios may have occurred. Junior ranking chiefs and second sons were leaving Tongatapu and going to outlying islands of the chiefdom, securing their power with intermarriage in the local ruling families and ensuring the prestige goods returned to Tongatapu [15]. While these ruling chiefs would not necessarily return to Tongatapu, there are ethnohistoric accounts of their retainers, possibly from Tongatapu themselves, returning to the sacred center of the chiefdom with prestige goods. Non-Tongans, representatives from other islands bringing tribute and trade goods to the Tu’i Tonga and his family, would also travel to Tongatapu. However, ethnohistoric accounts record the power of the maritime chiefdom and the sacredness of the island was such that these non-Tongans were not allowed to travel to Tongatapu without escort and not permitted to stay [41].

This highly mobile lifestyle was also hypothesized for the early Maori of Wairau Bar in New Zealand [81]. In the Wairau Bar assemblage, many individuals in burial groups 2/3 (the later burials) displayed ^87^Sr/^86^Sr values within the local mean (as determined from ^87^Sr/^86^Sr values from prehistoric Wairau Bar dogs). However, highly varied diets as interpreted through paleodietary isotopic analyses suggest that these people lived (and ate) in a variety of different locations before being buried at Wairau Bar. As evidenced at Wairau Bar, examining dietary differences are a powerful means of understanding mobility. A scenario parallel to that of Group 2/3 at Wairau Bar may have unfolded for some individuals interred in the `Atele burial mounds. To-At-2/33, with his δ^13^C values well outside the population mean, may have been born on Tongatapu, journeyed to other islands, and returned to Tongatapu a few years before his death. Unfortunately, with little material evidence in `Atele it is unclear who exactly would undergo these journeys and what sort of power and prestige they received when returning home to Tongatapu.

It is important to consider that the population ^87^Sr/^86^Sr mean (0.7089) is very different from the mean of the Tongan geological baseline (0.7037). Instead, the population mean is very similar to the mean ^87^Sr/^86^Sr isotopic signature of modern seawater (0.7092). It may be impossible to differentiate between small islands in West Polynesia using ^87^Sr/^86^Sr ratios. On relatively small islands such as Tongatapu (259 km^2^), sea spray would have constituted a large portion of bioavailable strontium. The relatively small islands comprising the archipelagoes of Tonga, Samoa, and much of East Polynesia could all have similar bioavailable ^87^Sr/^86^Sr reservoirs. The low ^87^Sr/^86^Sr ratio of To-At-1/09 may indicate this woman spent her childhood on a larger island such as Viti Levu, Fiji (only 800 km away and over 10,000 km^2^ in area). Until the biosphere signatures of Tongatapu and surrounding islands are characterized, using the population mean as a measure of locality is the only available option for interpretation.

As a final note, having only a single foreigner in the mound does not necessarily contrast with Clark et al.’s [31] conclusions of frequent contact and exchange. To-At-1/09 is 2.5% of the mortuary sample (1 of 41 individuals). If the `Atele burial mounds are demographically representative of the Tongatapu population during the Chiefdom period, and assuming 20,000 individuals lived on Tongatapu during the chiefdom period (the upper bounds of estimated pre-Contact population in the Tongan archipelago were 40,000 with most of the population on Tongatapu [12]), then the foreign population could be as high as 500 individuals. There are serious complications when trying to reconstruct the living profile of a community: a mortuary population can never be fully representative of the living population from whence it is derived [136,137]. In addition to the problems plaguing any cemetery sample, the `Atele burial mounds are particularly affected by sampling issues: as the main research focus of the original excavations was to determine the method of mound construction and types of mound use, neither mound was fully excavated [22]. Instead, trenches to the centers and on edges of the mounds were dug, and only 2.9% and 13.6% of the possible areas were excavated for the To-At-1 and To-At-2, respectively.

If there were a sizeable of immigrants from Samoa or Fiji living on Tonga, they might have lived together, as immigrants do today in the Pacific (such as the ‘Kapinga village on Pohnpei Island in the Federated States of Micronesia, where Polynesian migrants from Kapingamarangi Island form an insular community [138]). In this scenario, an as-of-yet unfound burial mound would inter the majority of non-locals on Tongatapu. Unfortunately, this is an untestable hypothesis using the current study’s data but is an intriguing possibility to consider nonetheless.

## Conclusion

This study used the isotopic analyses of bone collagen and enamel of individuals from Tongatapu to assess prehistoric diet and mobility during the Chiefdom Period. Dietary isotopic analyses indicated that those interred in the `Atele burial mounds consumed a diet consisting mostly of terrestrial C_3_ plants and marine animals, which corroborates with the ethnohistoric and archaeological evidence of Tongans consuming a diet mainly of starchy plants (e.g., yam, taro, and breadfruit) and marine foods (e.g., shellfish, reef and pelagic fish, sea turtles, and seaweed).

The dietary analysis indicates that those interred in each burial mound ate different proportions of foods, with To-At-2 individuals consuming more terrestrial plants. Our hypothesis regarding the differences in diet between those interred in the two burial mounds fails to be rejected. A possible cause for these dietary differences may be temporal, that the mounds were in use at different points of the Chiefdom Period. Another reason posited in this study is that the two mounds interred people of different status. The degree of status disparity between the two mounds is hard to define with any certainty as To-At-2 may not contain those of chiefly class, although perhaps higher-ranking commoners such as chiefly retainers or skilled workers were those interred here. Interpreting diet and how sex, status, and division of labor affect proportions of food types proved more complicated than anticipated in this study. Our hypothesis that males and females would consume significantly different proportions of foods fails to be rejected, but finding the underlying reasons for these differences was not possible. The positive correlation between δ^34^S values and wt %S may indicate post-mortem diagenetic alteration and may indicate that sulfur analysis is not an effective means to study paleodiet on some Pacific islands.

High levels of mobility in Tongatapu during this period of the maritime empire, if present, were not observed using isotopic analyses in this study. The strontium results indicate that the majority of the population was local and few immigrants came from other islands (at least those with discernibly different geological values). This contrasts with the archaeological evidence demonstrated by Clark et al. [31] of movement of prestige goods into and out of Tongatapu. Rather than contradicting the Clark et al [31] study, the same forces that affected the movement of goods may have affected the movement of people where the religiopolitical influence of the empire ensured the movement of tribute to Tongatapu, while that same control prevented permanent movement of outsiders to the island.

This study demonstrated that further isotopic analyses with this assemblage could yield substantive information about diet, subsistence, and the sociocultural influences affecting individual food choices. The interaction between childhood and social status is not well understood in prehistoric Polynesia. Tooth collagen has been collected from the same individuals who had teeth available for ^87^Sr/^86^Sr analysis. By comparing the dietary isotopic compositions of tooth collagen to bone collagen it may be possible to examine their childhood diet in order to compare their food consumption patterns at two different points in their life histories. This may provide information as to whether there are dietary distinctions based on age and whether status-related dietary differences were present during childhood.

Comparing oral health as an indicator of diet and the dietary findings from isotopic analyses is another avenue of future research. As the traditional Polynesian diet does not fall neatly into the “hunter-gatherer/agriculturalist” continuum commonly used in interpreting diet from oral health in other parts of the world [139–141] the comparison of dental health to isotopic analyses provides an opportunity to explore possible discrepancies between methods of paleodiet reconstruction.

## Supporting Information

S1 Table Paleodemographic information and isotopic analyses results for individuals from the `Atele site, sorted by burial number. (DOCX)
