# Supplementary material for: Assessing Human Diet and Movement in the Tongan Maritime Chiefdom Using Isotopic Analyses
Source: PLoS One. 2015 Mar 30;10(3):e0123156. doi: 10.1371/journal.pone.0123156 (PMC4378966; doi:10.1371/journal.pone.0123156)
Supplement: S1 Table — (DOCX) [file pone.0123156.s001.docx]

| **Burial Number** | **Age** | **Sex** | **Bone element** | **δ^13^C** | **δ^15^N** | **δ^34^S** | **^87^Sr^/86^Sr** | **%C** | **%N** | **%S** | **C:N** | **C:S** | **N:S** | **Sr conc. (ppm)** |
| --- | --- | --- | --- | --- | --- | --- | --- | --- | --- | --- | --- | --- | --- | --- |
| To-At-1/04a | UA | F | Long bone |  |  |  | 0.7087 |  |  |  |  |  |  | 175.8 |
| To-At-1/06 | MA | M | Humerus | -17.6 | 9.9 | 14.5 | 0.7089 | 39.8 | 14.9 | 0.24 | 3.1 | 447.4 | 143.7 | 276.6 |
| To-At-1/07 | MA | M | Tibia | -18.2 | 8.4 | 13.2 | 0.7088 | 37.9 | 13.9 | 0.25 | 3.2 | 410.0 | 129.0 | 192.9 |
| To-At-1/09 | YA | F | Scapula | -17.8 | 8.8 | 13.3 | 0.7085 | 39.8 | 14.3 | 0.26 | 3.3 | 408.3 | 125.5 | 166.4 |
| To-At-1/11 | YA | F | Humerus | -17.9 | 8.7 | 15.5 | 0.7088 | 39.3 | 14.2 | 0.26 | 3.2 | 409.2 | 127.0 | 261.2 |
| To-At-1/12 | MA | F | Long bone | -18.0 | 9.6 | 17.2 |  | 37.6 | 13.6 | 0.25 | 3.2 | 395.9 | 122.3 |  |
| To-At-1/13 | YA | F | Long bone | -17.6 | 8.1 | 13.2 |  | 42.2 | 15.3 | 0.23 | 3.2 | 487.7 | 152.0 |  |
| To-At-1/19 | YA | F | Long bone | -16.7 | 9.3 | 13.1 | 0.7089 | 43.4 | 15.9 | 0.21 | 3.2 | 557.9 | 175.1 | 229.5 |
| To-At-1/20 | YA | M | Humerus | -17.1 | 9.5 | 16.0 | 0.7088 | 37.4 | 13.8 | 0.24 | 3.2 | 413.7 | 130.6 | 127.9 |
| To-At-1/21a(1) | UA | F | Long bone | -17.0 | 9.7 | 15.5 | 0.7087 | 37.3 | 13.6 | 0.26 | 3.2 | 378.4 | 118.0 | 241.8 |
| To-At-1/21a(2) | UA | F | Femur |  |  |  | 0.7089 |  |  |  |  |  |  | 86.8 |
| To-At-1/21b | UA | F | Femur | -16.9 | 9.8 | 16.7 |  | 38.2 | 13.8 | 0.23 | 3.2 | 450.6 | 139.8 |  |
| To-At-1/23 | YA | F | Long bone | -17.1 | 9.0 | 14.5 | 0.7090 | 39.6 | 14.5 | 0.25 | 3.2 | 423.7 | 133.3 | 328.6 |
| To-At-1/26 | YA | F | Ulna | -18.4 | 8.7 |  | 0.7090 | 40.0 | 14.4 |  | 3.3 |  |  | 267.9 |
| To-At-1/27 | UA | M | Long bone | -16.2 | 9.6 | 12.4 |  | 40.0 | 14.2 | 0.22 | 3.3 | 477.5 | 145.1 |  |
| To-At-1/29a | MA | M | Long bone | -16.4 | 10.3 |  | 0.7086 | 42.7 | 15.7 |  | 3.2 |  |  | 206.6 |
| To-At-1/31 | MA | M | Long bone | -17.7 | 9.6 | 15.6 |  | 37.7 | 13.2 | 0.29 | 3.3 | 353.4 | 106.3 |  |
| To-At-1/34 | YA | M | Long bone | -16.8 | 9.5 |  | 0.7088 | 39.9 | 14.6 |  | 3.2 |  |  | 244.8 |
| To-At-2/01c | OA | F | Vertebral process | -17.5 | 10 | 13.6 | 0.7091 | 38.7 | 14.1 | 0.20 | 3.2 | 504.9 | 157.1 | 196.3 |
| To-At-2/01e(2) | YA | F | Long bone |  |  |  | 0.7089 |  |  |  |  |  |  | 122.4 |
| To-At-2/04 | MA | M | Fibula | -18.2 | 9.1 | 15.7 |  | 42.0 | 15.5 | 0.27 | 3.2 | 420.9 | 133.4 |  |
| To-At-2/06 | MA | M | Scapula | -18.2 | 8.5 | 15.1 | 0.7086 | 40.5 | 14.5 | 0.27 | 3.3 | 401.6 | 123.4 | 145.6 |
| To-At-2/08 | UA | M | Tibia | -16.8 | 10.6 | 16.1 |  | 40.4 | 14.9 | 0.24 | 3.2 | 443.4 | 140.1 |  |
| To-At-2/11 | YA | F | Humerus | -18.5 | 8.4 | 16.0 |  | 39.9 | 13.8 | 0.28 | 3.4 | 380.8 | 112.9 |  |
| To-At-2/13 | OA | M | Humerus | -18.1 | 9.8 | 14.8 |  | 41.9 | 15.1 | 0.26 | 3.2 | 431.3 | 133.4 |  |
| To-At-2/13a | MA | M | Humerus | -17.3 | 9.8 | 14.8 | 0.7088 | 41.7 | 14.9 | 0.23 | 3.3 | 475.7 | 145.5 | 111.2 |
| To-At-2/13b | OA | F | Tibia | -18.0 | 9.3 | 13.2 | 0.7088 | 38.6 | 14.0 | 0.26 | 3.2 | 395.7 | 122.5 | 346 |
| To-At-2/16 | UA | F | Ulna | -17.9 | 8.6 | 12.3 | 0.7087 | 38.2 | 13.8 | 0.23 | 3.2 | 437.3 | 135.4 | 129.1 |
| To-At-2/18 | OA | M | Scapula | -17.2 | 10.3 | 14.4 |  | 37.3 | 13.4 | 0.28 | 3.3 | 359.3 | 110.3 |  |
| To-At-2/20a | UA | F | Femur | -18 | 8.7 | 14.6 |  | 41.5 | 15.7 | 0.24 | 3.1 | 466.7 | 151.8 |  |
| To-At-2/21 | YA | F | Long bone | -18.1 | 9.1 | 13.7 |  | 42.4 | 15.2 | 0.25 | 3.3 | 446.1 | 137.0 |  |
| To-At-2/24a | YA | F | Long bone | -18.1 | 8.8 | 14.8 | 0.7089 | 38.1 | 14.0 | 0.21 | 3.2 | 475.4 | 149.6 | 212.8 |
| To-At-2/24b | OA | F | Humerus | -18.0 | 9.1 | 13.1 | 0.7087 | 40.6 | 14.8 | 0.24 | 3.2 | 449.5 | 140.5 | 208.9 |
| To-At-2/25 | OA | F | Scapula | -17.9 | 8.6 | 13.9 | 0.7089 | 42.5 | 15.0 | 0.26 | 3.3 | 438.2 | 132.8 | 223.9 |
| To-At-2/27 | YA | F | Tibia | -18.2 | 8.6 | 15.4 | 0.7088 | 41.3 | 15.1 | 0.20 | 3.2 | 537.8 | 169.3 | 246.7 |
| To-At-2/27a | MA | M | Tibia | -17.4 | 8.6 | 9.9 |  | 38.3 | 14.2 | 0.22 | 3.1 | 474.7 | 150.8 |  |
| To-At-2/30 | UA | F | Tiba | -18.0 | 9.4 | 14.7 | 0.7089 | 41.3 | 14.6 | 0.26 | 3.3 | 428.5 | 129.9 | 203.9 |
| To-At-2/31 | YA | M | Long bone | -18.4 | 9.3 |  | 0.7090 | 41.6 | 14.9 |  | 3.2 |  |  | 253 |
| To-At-2/32 | UA | F | Femur | -18.5 | 9.3 | 16.4 | 0.7088 | 38.6 | 14.0 | 0.24 | 3.2 | 426.1 | 132.1 | 299 |
| To-At-2/33 | YA | M | Tiba | -14.8 | 9.7 | 17.2 | 0.7089 | 36.2 | 13.3 | 0.28 | 3.2 | 340.4 | 107.7 | 291.8 |
| To-At-2/34 | UA | M | Long bone | -18.4 | 9.2 | 13.7 |  | 41.2 | 15.3 | 0.23 | 3.1 | 469.6 | 149.8 |  |
| To-At-2/40a | MA | F | Long bone | -18.1 | 8.8 |  | 0.7089 | 43.6 | 15.7 |  | 3.2 |  |  | 178.9 |
| To-At-2/40b | MA | F | Ulna |  |  |  | 0.7088 |  |  |  |  |  |  | 222.8 |
| To-At-2/41a | OA | M | Ulna | -17.7 | 9.9 | 11.6 |  | 41.5 | 15.2 | 0.24 | 3.2 | 464.7 | 145.8 |  |
| To-At-2/42 | YA | F | Ulna | -18.1 | 9.4 | 17.8 | 0.7089 | 41.5 | 14.9 | 0.26 | 3.3 | 430.8 | 132.3 | 306.6 |

NB: For age, YA= Young Adult, MA= Middle-aged adult, OA= old adult, UA= Adult of indeterminate age

“Long bone” elements were cortical bone fragments from unidentifiable long bones.
